# 4-{Eth­yl[(*E*)-4-(4-pyridylvin­yl)phenyl]­amino}benzaldehyde

**DOI:** 10.1107/S1600536808031358

**Published:** 2008-10-04

**Authors:** Dao-Fu Liu, Yong-Hong Chen, Feng-Wu Wang

**Affiliations:** a Department of Chemistry, Huainan Normal University, Huainan 232001, People’s Republic of China; b Department of Chemistry, Anhui University, Hefei 230039, People’s Republic of China

## Abstract

In the title mol­ecule, C_22_H_20_N_2_O, the central aromatic ring forms dihedral angles of 45.30 (2) and 69.43 (2)°, respectively, with the outer pyridine and benzene rings. In the crystal structure, weak inter­molecular C—H⋯O inter­actions link the mol­ecules into layers parallel to the *ab* plane.

## Related literature

For related structure information, see: Allen *et al.* (1987 ▶). For general background, see: Marder (2006 ▶).
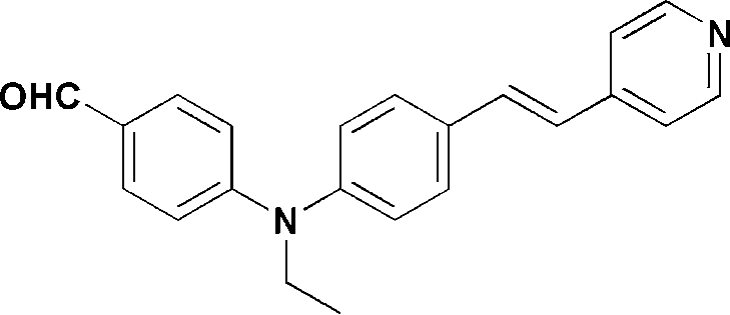


## Experimental

### 

#### Crystal data

C_22_H_20_N_2_O
*M* *_r_* = 328.40
Triclinic, 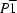

*a* = 8.8338 (14) Å
*b* = 9.5747 (18) Å
*c* = 10.472 (2) Å
α = 86.621 (2)°
β = 84.276 (1)°
γ = 83.886 (1)°
*V* = 875.3 (3) Å^3^
*Z* = 2
Mo *K*α radiation
μ = 0.08 mm^−1^
*T* = 298 (2) K
0.50 × 0.40 × 0.36 mm

#### Data collection

Bruker SMART area-dectector diffractometer
Absorption correction: multi-scan (*SADABS*; Bruker, 2002 ▶) *T*_min_ = 0.963, *T*_max_ = 0.973
4562 measured reflections
3036 independent reflections
2092 reflections with *I* > 2σ(*I*)
*R*_int_ = 0.023

#### Refinement

*R*[*F* ^2^ > 2σ(*F* ^2^)] = 0.067
*wR*(*F* ^2^) = 0.209
*S* = 1.02
3036 reflections
228 parameters
H-atom parameters constrained
Δρ_max_ = 0.59 e Å^−3^
Δρ_min_ = −0.21 e Å^−3^

### 

Data collection: *SMART* (Bruker, 2002 ▶); cell refinement: *SAINT* (Bruker, 2002 ▶); data reduction: *SAINT*; program(s) used to solve structure: *SHELXS97* (Sheldrick, 2008 ▶); program(s) used to refine structure: *SHELXL97* (Sheldrick, 2008 ▶); molecular graphics: *ORTEPII* (Johnson, 1976 ▶); software used to prepare material for publication: *SHELXL97*.

## Supplementary Material

Crystal structure: contains datablocks I, global. DOI: 10.1107/S1600536808031358/cv2455sup1.cif

Structure factors: contains datablocks I. DOI: 10.1107/S1600536808031358/cv2455Isup2.hkl

Additional supplementary materials:  crystallographic information; 3D view; checkCIF report

## Figures and Tables

**Table 1:** Hydrogen-bond geometry (Å, °)

*D*—H⋯*A*	*D*—H	H⋯*A*	*D*⋯*A*	*D*—H⋯*A*
C21—H21*B*⋯O1^i^	0.97	2.60	3.384 (4)	138
C15—H15⋯O1^ii^	0.93	2.63	3.553 (4)	175

Symmetry codes: (i) 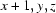; (ii) 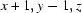.

**Fig. 1.**
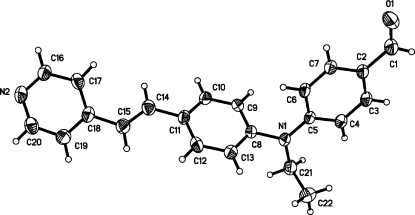
The molecular structure of the title compound showing 30% probability displacement ellipsoids.

## supplementary crystallographic information

### Comment

Nonlinear optical (NLO) organic materials have been extensively studied due to their broad applications in the area of electronics and photonics (Marder, 2006). It has been found that the delocalized conjugated electrons contribute to enhancing the NLO response through their capability for hyperpolarization. As a part of our ongoing investigation of NLO materials, the title compound has been prepared. Its crystal structure is presented here.

The molecular structure of the title compound is shown in Fig. 1. Bond lengths and angles in the molecule are in agreement with the values reported in the literature (Allen *et al.*, 1987). The C11—C14 and C15—C18 bond lengths are indicative of double-bond character. Therefore, there is a high electron delocalization in the π-system of the molecule. The dihedral angle formed by the pyridine ring and C8—C13 benzene ring is 45.30 (2)°.

In the crystal, weak intermolecular C—H···O interactions (Table 1) link the molecules into layers parallel to *ab* plane.

### Experimental

For the preparation of 4-(*N*-ethyl-N-(4-((E)-2-(pyridin-4-yl) vinyl)phenyl)amino)benzaldehyde: A mixture of 4-(*N*-ethyl-*N*-(4-iodophenyl)amino)benzaldehyde (3.15 g, 10 mmol), Pd(OAc)_2_ (0.0330 g), triethylamine (15 ml) and 4-vinylpyridine (15 ml) were heated at 363 K with CH_3_CN (40 ml) as solvent for 40 h under nitrogen. The mixture was cooled to room temperature and added to 500 ml water. Plentiful yellow solid was obtained by filtration. This was dissolved in dichloromethane. The solution was washed twice with water, dried over anhydrous magnesium sulfate, then filtered and concentrated. The resulting solution was purified by flash column chromatography with dichloromethane as eluent to give light yellow crystals (1.95 mg, yield 56%).

### Refinement

All hydrogen atoms were placed in geometrically idealized positions and constrained to ride on their parent atoms, with C—H = 0.93–0.97 Å and *U*_iso_(H) = 1.2 or 1.5 *U*_eq_(C).

### Crystal data

**Table:**

C_22_H_20_N_2_O	*Z* = 2
*M**_r_* = 328.40	*F*(000) = 348
Triclinic, *P*1	*D*_x_ = 1.246 Mg m^−^^3^
*a* = 8.8338 (14) Å	Mo *K*α radiation, λ = 0.71073 Å
*b* = 9.5747 (18) Å	Cell parameters from 1751 reflections
*c* = 10.472 (2) Å	θ = 2.3–27.2°
α = 86.621 (2)°	µ = 0.08 mm^−^^1^
β = 84.276 (1)°	*T* = 298 K
γ = 83.886 (1)°	Block, yellow
*V* = 875.3 (3) Å^3^	0.50 × 0.40 × 0.36 mm

### Data collection

**Table:**

Bruker APEX area-dectector diffractometer	3036 independent reflections
Radiation source: fine-focus sealed tube	2092 reflections with *I* > 2σ(*I*)
graphite	*R*_int_ = 0.023
φ and ω scans	θ_max_ = 25.0°, θ_min_ = 2.1°
Absorption correction: multi-scan (SADABS; Bruker, 2002)	*h* = −10→10
*T*_min_ = 0.963, *T*_max_ = 0.973	*k* = −11→11
4562 measured reflections	*l* = −12→9

### Refinement

**Table:**

Refinement on *F*^2^	Secondary atom site location: difference Fourier map
Least-squares matrix: full	Hydrogen site location: inferred from neighbouring sites
*R*[*F*^2^ > 2σ(*F*^2^)] = 0.067	H-atom parameters constrained
*wR*(*F*^2^) = 0.210	*w* = 1/[σ^2^(*F*_o_^2^) + (0.0993*P*)^2^ + 0.5822*P*] where *P* = (*F*_o_^2^ + 2*F*_c_^2^)/3
*S* = 1.02	(Δ/σ)_max_ < 0.001
3036 reflections	Δρ_max_ = 0.59 e Å^−^^3^
228 parameters	Δρ_min_ = −0.21 e Å^−^^3^
0 restraints	Extinction correction: SHELXL97 (Sheldrick, 2008), Fc^*^=kFc[1+0.001xFc^2^λ^3^/sin(2θ)]^-1/4^
Primary atom site location: structure-invariant direct methods	Extinction coefficient: 0.053 (9)

### Special details

**Table:**

Geometry. All e.s.d.'s (except the e.s.d. in the dihedral angle between two l.s. planes) are estimated using the full covariance matrix. The cell e.s.d.'s are taken into account individually in the estimation of e.s.d.'s in distances, angles and torsion angles; correlations between e.s.d.'s in cell parameters are only used when they are defined by crystal symmetry. An approximate (isotropic) treatment of cell e.s.d.'s is used for estimating e.s.d.'s involving l.s. planes.
Refinement. Refinement of *F*^2^ against ALL reflections. The weighted *R*-factor *wR* and goodness of fit *S* are based on *F*^2^, conventional *R*-factors *R* are based on *F*, with *F* set to zero for negative *F*^2^. The threshold expression of *F*^2^ > σ(*F*^2^) is used only for calculating *R*-factors(gt) *etc*. and is not relevant to the choice of reflections for refinement. *R*-factors based on *F*^2^ are statistically about twice as large as those based on *F*, and *R*- factors based on ALL data will be even larger.

### Fractional atomic coordinates and isotropic or equivalent isotropic displacement parameters (Å^2^)

**Table:**

	*x*	*y*	*z*	*U*_iso_*/*U*_eq_	
N1	0.6686 (3)	0.5962 (2)	0.8163 (2)	0.0589 (7)	
N2	0.8199 (3)	−0.3738 (3)	0.3196 (3)	0.0651 (7)	
O1	−0.0027 (3)	0.9357 (3)	0.8369 (3)	0.0855 (8)	
C1	0.1224 (4)	0.9637 (3)	0.8579 (3)	0.0670 (9)	
H1	0.1299	1.0540	0.8831	0.080*	
C2	0.2628 (3)	0.8680 (3)	0.8471 (3)	0.0543 (7)	
C3	0.4016 (4)	0.9124 (3)	0.8714 (3)	0.0637 (9)	
H3	0.4035	1.0041	0.8956	0.076*	
C4	0.5361 (4)	0.8255 (3)	0.8609 (3)	0.0610 (8)	
H4	0.6269	0.8589	0.8774	0.073*	
C5	0.5362 (3)	0.6866 (3)	0.8254 (3)	0.0502 (7)	
C6	0.3954 (3)	0.6420 (3)	0.8015 (3)	0.0503 (7)	
H6	0.3921	0.5501	0.7787	0.060*	
C7	0.2630 (3)	0.7309 (3)	0.8109 (2)	0.0509 (7)	
H7	0.1720	0.6989	0.7928	0.061*	
C8	0.6755 (3)	0.4681 (3)	0.7520 (3)	0.0502 (7)	
C9	0.6535 (3)	0.4731 (3)	0.6235 (3)	0.0496 (7)	
H9	0.6291	0.5594	0.5810	0.060*	
C10	0.6671 (3)	0.3521 (3)	0.5574 (3)	0.0522 (7)	
H10	0.6499	0.3577	0.4710	0.063*	
C11	0.7055 (3)	0.2228 (3)	0.6162 (3)	0.0518 (7)	
C12	0.7281 (4)	0.2186 (3)	0.7447 (3)	0.0635 (8)	
H12	0.7544	0.1324	0.7867	0.076*	
C13	0.7127 (4)	0.3398 (3)	0.8128 (3)	0.0633 (8)	
H13	0.7275	0.3343	0.8997	0.076*	
C14	0.7174 (3)	0.0976 (3)	0.5376 (3)	0.0604 (8)	
H14	0.6689	0.1084	0.4622	0.073*	
C15	0.7878 (3)	−0.0247 (3)	0.5623 (3)	0.0611 (8)	
H15	0.8373	−0.0381	0.6371	0.073*	
C16	0.7031 (4)	−0.2750 (3)	0.3192 (3)	0.0608 (8)	
H16	0.6279	−0.2837	0.2649	0.073*	
C17	0.6865 (3)	−0.1606 (3)	0.3941 (3)	0.0600 (8)	
H17	0.6028	−0.0936	0.3882	0.072*	
C18	0.7943 (3)	−0.1442 (3)	0.4789 (3)	0.0545 (7)	
C19	0.9135 (3)	−0.2486 (3)	0.4816 (3)	0.0643 (8)	
H19	0.9882	−0.2458	0.5378	0.077*	
C20	0.9211 (4)	−0.3565 (3)	0.4006 (3)	0.0676 (9)	
H20	1.0050	−0.4238	0.4027	0.081*	
C21	0.8183 (4)	0.6392 (3)	0.8480 (3)	0.0650 (8)	
H21A	0.9010	0.5750	0.8106	0.078*	
H21B	0.8313	0.7327	0.8102	0.078*	
C22	0.8276 (5)	0.6394 (4)	0.9886 (4)	0.0863 (11)	
H22A	0.7473	0.7045	1.0257	0.129*	
H22B	0.9249	0.6671	1.0049	0.129*	
H22C	0.8165	0.5467	1.0262	0.129*	

### Atomic displacement parameters (Å^2^)

**Table:**

	*U*^11^	*U*^22^	*U*^33^	*U*^12^	*U*^13^	*U*^23^
N1	0.0578 (15)	0.0496 (14)	0.0732 (16)	0.0037 (11)	−0.0218 (12)	−0.0252 (12)
N2	0.0703 (17)	0.0542 (16)	0.0700 (17)	−0.0036 (13)	0.0055 (14)	−0.0210 (13)
O1	0.0712 (16)	0.0721 (16)	0.112 (2)	0.0133 (13)	−0.0176 (14)	−0.0154 (14)
C1	0.078 (2)	0.0516 (18)	0.071 (2)	0.0061 (16)	−0.0103 (17)	−0.0125 (15)
C2	0.0678 (19)	0.0442 (16)	0.0501 (15)	0.0073 (13)	−0.0110 (13)	−0.0101 (12)
C3	0.083 (2)	0.0386 (16)	0.072 (2)	0.0053 (15)	−0.0229 (17)	−0.0201 (14)
C4	0.0686 (19)	0.0464 (17)	0.0721 (19)	−0.0005 (14)	−0.0229 (16)	−0.0194 (14)
C5	0.0638 (18)	0.0415 (15)	0.0471 (15)	0.0014 (13)	−0.0145 (13)	−0.0127 (11)
C6	0.0606 (17)	0.0398 (15)	0.0516 (15)	−0.0025 (12)	−0.0052 (13)	−0.0142 (12)
C7	0.0568 (17)	0.0490 (16)	0.0468 (15)	−0.0011 (13)	−0.0048 (12)	−0.0100 (12)
C8	0.0500 (15)	0.0428 (15)	0.0591 (17)	0.0025 (12)	−0.0129 (13)	−0.0148 (12)
C9	0.0467 (15)	0.0452 (15)	0.0567 (16)	0.0033 (12)	−0.0089 (12)	−0.0077 (12)
C10	0.0503 (16)	0.0568 (18)	0.0496 (15)	0.0012 (13)	−0.0050 (12)	−0.0145 (13)
C11	0.0479 (15)	0.0506 (17)	0.0574 (17)	−0.0021 (12)	−0.0007 (12)	−0.0187 (13)
C12	0.073 (2)	0.0423 (16)	0.073 (2)	0.0076 (14)	−0.0090 (16)	−0.0042 (14)
C13	0.082 (2)	0.0547 (18)	0.0540 (17)	0.0067 (16)	−0.0181 (15)	−0.0105 (14)
C14	0.0536 (17)	0.0574 (19)	0.0694 (19)	−0.0020 (14)	0.0002 (14)	−0.0120 (15)
C15	0.0547 (17)	0.061 (2)	0.0684 (19)	−0.0023 (15)	−0.0059 (15)	−0.0121 (15)
C16	0.0619 (19)	0.0567 (18)	0.0651 (18)	−0.0082 (15)	−0.0029 (15)	−0.0167 (15)
C17	0.0506 (17)	0.0494 (17)	0.077 (2)	0.0035 (13)	0.0046 (15)	−0.0106 (15)
C18	0.0506 (16)	0.0512 (17)	0.0626 (17)	−0.0125 (13)	0.0054 (13)	−0.0165 (13)
C19	0.0490 (17)	0.071 (2)	0.073 (2)	−0.0028 (15)	−0.0025 (14)	−0.0155 (16)
C20	0.0600 (19)	0.060 (2)	0.078 (2)	0.0078 (15)	0.0071 (16)	−0.0132 (17)
C21	0.0648 (19)	0.0600 (19)	0.071 (2)	−0.0030 (15)	−0.0064 (15)	−0.0157 (15)
C22	0.098 (3)	0.084 (3)	0.081 (2)	−0.003 (2)	−0.033 (2)	−0.012 (2)

### Geometric parameters (Å, °)

**Table:**

N1—C5	1.378 (3)	C11—C12	1.377 (4)
N1—C8	1.426 (3)	C11—C14	1.483 (4)
N1—C21	1.500 (4)	C12—C13	1.385 (4)
N2—C20	1.321 (4)	C12—H12	0.9300
N2—C16	1.323 (4)	C13—H13	0.9300
O1—C1	1.208 (4)	C14—C15	1.291 (4)
C1—C2	1.461 (4)	C14—H14	0.9300
C1—H1	0.9300	C15—C18	1.473 (4)
C2—C7	1.388 (4)	C15—H15	0.9300
C2—C3	1.391 (4)	C16—C17	1.372 (4)
C3—C4	1.374 (4)	C16—H16	0.9300
C3—H3	0.9300	C17—C18	1.391 (4)
C4—C5	1.402 (4)	C17—H17	0.9300
C4—H4	0.9300	C18—C19	1.374 (4)
C5—C6	1.408 (4)	C19—C20	1.367 (4)
C6—C7	1.370 (4)	C19—H19	0.9300
C6—H6	0.9300	C20—H20	0.9300
C7—H7	0.9300	C21—C22	1.482 (5)
C8—C13	1.373 (4)	C21—H21A	0.9700
C8—C9	1.375 (4)	C21—H21B	0.9700
C9—C10	1.373 (4)	C22—H22A	0.9600
C9—H9	0.9300	C22—H22B	0.9600
C10—C11	1.375 (4)	C22—H22C	0.9600
C10—H10	0.9300		
			
C5—N1—C8	120.6 (2)	C11—C12—H12	119.3
C5—N1—C21	122.0 (2)	C13—C12—H12	119.3
C8—N1—C21	116.3 (2)	C8—C13—C12	120.0 (3)
C20—N2—C16	115.0 (3)	C8—C13—H13	120.0
O1—C1—C2	125.7 (3)	C12—C13—H13	120.0
O1—C1—H1	117.2	C15—C14—C11	127.0 (3)
C2—C1—H1	117.2	C15—C14—H14	116.5
C7—C2—C3	117.8 (3)	C11—C14—H14	116.5
C7—C2—C1	121.4 (3)	C14—C15—C18	123.7 (3)
C3—C2—C1	120.8 (3)	C14—C15—H15	118.1
C4—C3—C2	122.3 (3)	C18—C15—H15	118.1
C4—C3—H3	118.9	N2—C16—C17	123.9 (3)
C2—C3—H3	118.9	N2—C16—H16	118.1
C3—C4—C5	120.0 (3)	C17—C16—H16	118.1
C3—C4—H4	120.0	C16—C17—C18	120.3 (3)
C5—C4—H4	120.0	C16—C17—H17	119.8
N1—C5—C4	121.4 (3)	C18—C17—H17	119.8
N1—C5—C6	121.1 (2)	C19—C18—C17	115.8 (3)
C4—C5—C6	117.5 (3)	C19—C18—C15	119.7 (3)
C7—C6—C5	121.6 (3)	C17—C18—C15	124.5 (3)
C7—C6—H6	119.2	C20—C19—C18	119.2 (3)
C5—C6—H6	119.2	C20—C19—H19	120.4
C6—C7—C2	120.8 (3)	C18—C19—H19	120.4
C6—C7—H7	119.6	N2—C20—C19	125.8 (3)
C2—C7—H7	119.6	N2—C20—H20	117.1
C13—C8—C9	118.8 (3)	C19—C20—H20	117.1
C13—C8—N1	121.8 (3)	C22—C21—N1	112.0 (3)
C9—C8—N1	119.3 (3)	C22—C21—H21A	109.2
C10—C9—C8	120.7 (3)	N1—C21—H21A	109.2
C10—C9—H9	119.6	C22—C21—H21B	109.2
C8—C9—H9	119.6	N1—C21—H21B	109.2
C9—C10—C11	121.4 (3)	H21A—C21—H21B	107.9
C9—C10—H10	119.3	C21—C22—H22A	109.5
C11—C10—H10	119.3	C21—C22—H22B	109.5
C10—C11—C12	117.6 (3)	H22A—C22—H22B	109.5
C10—C11—C14	117.8 (3)	C21—C22—H22C	109.5
C12—C11—C14	124.6 (3)	H22A—C22—H22C	109.5
C11—C12—C13	121.5 (3)	H22B—C22—H22C	109.5
			
O1—C1—C2—C7	−0.4 (5)	C9—C10—C11—C12	0.8 (4)
O1—C1—C2—C3	178.8 (3)	C9—C10—C11—C14	179.9 (3)
C7—C2—C3—C4	−0.2 (5)	C10—C11—C12—C13	0.1 (5)
C1—C2—C3—C4	−179.4 (3)	C14—C11—C12—C13	−178.9 (3)
C2—C3—C4—C5	−0.4 (5)	C9—C8—C13—C12	0.3 (5)
C8—N1—C5—C4	−165.1 (3)	N1—C8—C13—C12	−175.7 (3)
C21—N1—C5—C4	2.2 (4)	C11—C12—C13—C8	−0.7 (5)
C8—N1—C5—C6	15.9 (4)	C10—C11—C14—C15	160.8 (3)
C21—N1—C5—C6	−176.8 (3)	C12—C11—C14—C15	−20.1 (5)
C3—C4—C5—N1	−178.9 (3)	C11—C14—C15—C18	180.0 (3)
C3—C4—C5—C6	0.1 (4)	C20—N2—C16—C17	1.2 (5)
N1—C5—C6—C7	179.7 (3)	N2—C16—C17—C18	−1.3 (5)
C4—C5—C6—C7	0.7 (4)	C16—C17—C18—C19	−0.3 (4)
C5—C6—C7—C2	−1.3 (4)	C16—C17—C18—C15	179.3 (3)
C3—C2—C7—C6	1.0 (4)	C14—C15—C18—C19	154.8 (3)
C1—C2—C7—C6	−179.8 (3)	C14—C15—C18—C17	−24.9 (5)
C5—N1—C8—C13	−122.4 (3)	C17—C18—C19—C20	1.9 (4)
C21—N1—C8—C13	69.6 (4)	C15—C18—C19—C20	−177.8 (3)
C5—N1—C8—C9	61.7 (4)	C16—N2—C20—C19	0.5 (5)
C21—N1—C8—C9	−106.4 (3)	C18—C19—C20—N2	−2.1 (5)
C13—C8—C9—C10	0.6 (4)	C5—N1—C21—C22	76.9 (4)
N1—C8—C9—C10	176.6 (2)	C8—N1—C21—C22	−115.3 (3)
C8—C9—C10—C11	−1.1 (4)		

### Hydrogen-bond geometry (Å, °)

**Table:**

*D*—H···*A*	*D*—H	H···*A*	*D*···*A*	*D*—H···*A*
C21—H21B···O1^i^	0.97	2.60	3.384 (4)	138
C15—H15···O1^ii^	0.93	2.63	3.553 (4)	175

Symmetry codes: (i) *x*+1, *y*, *z*; (ii) *x*+1, *y*−1, *z*.
